# Current Status and Trends in Alternative Models to Study Fungal Pathogens

**DOI:** 10.3390/jof5010012

**Published:** 2019-01-26

**Authors:** Juliana Campos Junqueira, Eleftherios Mylonakis

**Affiliations:** 1 Department of Biosciences and Oral Diagnosis, Institute of Science and Technology, São Paulo State University/UNESP, São José dos Campos, SP 12245-000, Brazil; 2 Division of Infectious Diseases, Rhode Island Hospital, Alpert Medical School of Brown University, Providence, RI 02903, USA

Fungal infections affect over a billion people, with mortality rates estimated at 1–2 million per year [1,2,3]. Advances in modern medicine, such as solid organ and bone marrow transplants, or anti-neoplasic chemotherapy, and the AIDS pandemic are associated with the dramatic increases of opportunistic fungal infections [1,4,5,6]. However, antifungal options for the treatment systemic fungal diseases are still limited [3]. To investigate the fungal–host interactions and to discover new antifungal agents, it is essential to use a variety of in vivo models [7,8]. Over the last decade, it has become clear that non-mammalian model hosts can be very useful for validating in vitro results and to test hypotheses that could be translated into higher organisms and clinical studies [9,10,11]. In this special issue, invertebrate, zebrafish, and amoeboid models are carefully discussed to provide an update on recent advances and a reflection on future directions.

Pereira et al. [12] present a detailed discussion on *Galleria mellonella*, that is one of the most common insect invertebrate models used in biomedical research. *G. mellonella* is an insect with functional similarities to the innate immune response of mammals. In this review, the authors outline the immune response of *G. mellonella* with a focus on the cellular process of phagocytosis, nodulation, and encapsulation, as well as other responses, such as antimicrobial peptides (AMPs), melanization, clotting, free radical production, and primary immunization. The main advances in the *G. mellonella* model are also discussed, including the sequencing of its genome and the availability of transcriptomic data. Notably, recent results from transcriptomic analyses reveal that the immune system of *G. mellonella* can distinguish between specific classes of pathogens.

Extending the Pereira et al. [12] report, Zaragoza and coworkers [13] discuss how the immune components of *G. mellonella* respond to infection by different fungal pathogens. For example, after infection by *Candida albicans*, the hemocytes of *G. mellonella* induce nodulation around *C. albicans* cells and this process is accompanied by early melanization. The immune response to *Aspergillus fumigatus* also results in the formation of melanized nodules and accumulation of antimicrobial peptides. Interestingly, infection with non-lethal inocula of *C. albicans* or *A. fumigatus* is enough to protect the larvae against a subsequent infection with lethal inocula. Interestingly, in the case of *Cryptococcus neoformans*, the infection in *G. mellonella* is not accompanied by early melanization due to the presence of the capsule and to the different cell wall composition. In the infection by *C. neoformans*, fungal cells are avidly phagocytosed by *G. mellonella* hemocytes, but can survive and replicate after internalization, behaving as intracellular pathogens similar to infection processes in mammals. Moreover, Singulani et al. [14] highlight specific aspects of immune response of *G. mellonella* against other important fungal pathogens, including *Paracoccidioides* spp., *Histoplasma capsulation*, *Blastomyces dermatitidis*, *Sporothrix* spp, and *Talaromyces marneffei*.

Given that several fungal infections can be studied in *G. mellonella*, this model creates the possibility of determining the efficacy of antifungal agents by evaluating both larval survival, as well as by monitoring the immune response associated with the infection process [15]. In this context, Kavanagh and Sheehan [15] explore the use of the *G. mellonella* model for the study of antifungal treatments. Many topics are discussed in this article, such as the assays to test relative toxicity in vivo, to identify the optimum combinations of antifungal drugs for resistant microorganisms, to study the mode of action of different classes of compounds, and to establish dosages prior to use in mammals. In another article, Cruz et al. [16] show inedited results of their research with four thiazolylhdrazone derivatives. The compounds tested demonstrated low toxicity, were able to prolong the survival of *G. mellonella* larvae infected with *C. albicans,* and reached the same efficacy as fluconazole. Among them, compound 2 (2-((2-(hexan-3-ylidene) hydrazino)-4-(4-methoxyphenyl)-thiazole) showed the most promising results and was identified as a potential candidate for further studies in murine models.

The *G. mellonella* model has been widely adopted by many researchers around the world [17,18,19,20,21,22]. However, the lack of standardization of larvae quality impairs the reproducibility and comparison of data between laboratories [17,23,24]. In this special issue, Champion et al. [23] raise an interesting discussion about the factors that need to be considered during the handling of *G. mellonella* for research, including age, size, availability of food, reared techniques, physical stress to transportation, incubation temperature, storage time, and possible residual levels of antibiotics and hormones. The authors suggest solutions on how to address these issues such as the pathological scoring system using automated real-time imaging, and developing of a standard well-characterized *G. mellonella* strain for research. While research and standardized procedures advance rapidly to make *G. mellonella* a robust and consistent infection model [23], other insect models can also be explored. For example, Souza et al. [25] present *Tenebrio molitor* (mealworm) as a potential model for the study of pathogenic fungi. This insect host has similar characteristics to *G. mellonella*, however its breeding in laboratory conditions is less laborious. These authors outline the first studies with *T. molitor* involving *C. albicans*, *C. neoformans*, *Malassezia furfur* and *Fonsecaea pedrosi*, and discuss the advantages of this model for the study of fungal virulence, mycotoxin effects, host immune responses, and the efficacy of antifungal agents.

In addition to insect model hosts, the nematode *Caenorhabditis elegans* is approached by Elkabti et al. [26] who describe the recent advances of this model to study *Candida* spp. infections. A great focus is given to the development of new visualization techniques to enhance capturing infection processes within live worms, tracking of nematodes, and the automation of high throughput assays. Other interesting topics are also discussed, such as the recent studies on the microbiome of *C. elegans* in nature, and the use of this model to identify molecular mechanisms involved in the development of drug resistance. In another article, Rosowski et al. [27] present an extensive review on the zebrafish model, a vertebrate animal. These authors emphasize the unique advantages of this model for the study of host–pathogen interactions. The zebrafish model offers multiple routes for infection with the possibility to monitor the infection process through the whole-body and at different localizations. In this way, recent advances on the development of live imaging platforms are discussed, such as light sheet fluorescence microscopy (LSFM) and correlative light and electron microscopy (CLEM).

To complete this special issue, Casadevall et al. raise important aspects in relation to *Acanthamoeba* spp., a free-living protozoa also used as a model host for fungal pathogens [28]. The article highlights the correlation of virulence factors between animal models and amoeba, and discusses the concept that animal pathogenic fungi acquire many of their virulence characteristics from interactions with environmental predators, such as soli amoeboid. Amoeba predation can be also associated with metabolic changes that reduce fungal susceptibility to polyenes. The authors highlight the importance to consider that mammals are only a small fraction of any environmental niche, and that many virulence attributes are a result of fungal interactions with other hosts.

Overall this special issue highlights the consensus regarding the alternative model hosts, such as amoebae, insects, worms, and zebrafish that are valuable and reliable tools to study fungal pathogenesis, but there are still several challenges and unexplored fields to be conquered.
